# Sugar transporters of the SWEET family and their role in arbuscular mycorrhiza

**DOI:** 10.18699/VJ21.086

**Published:** 2021-11

**Authors:** A.A. Kryukov, A.O. Gorbunova, T.R. Kudriashova, O.I. Yakhin, A.A. Lubyanov, U.M. Malikov, M.F. Shishova, A.P. Kozhemyakov, A.P. Yurkov

**Affiliations:** All-Russian Research Institute for Agricultural Microbiology, Pushkin, St. Petersburg, Russia; All-Russian Research Institute for Agricultural Microbiology, Pushkin, St. Petersburg, Russia; Peter the Great St. Petersburg Polytechnic University, St. Petersburg, Russia; Institute of Biochemistry and Genetics – Subdivision of the Ufa Federal Research Center of the Russian Academy of Sciences, Ufa, Russia; Tatar Research Institute of Agricultural Chemistry and Soil Science – Subdivision of the Federal Research Center “Kazan Scientif ic Center of the Russian Academy of Sciences”, Kazan, Russia; Research, Development and Production Enterprise “Eco Priroda”, Ulkundy village, Duvansky district, Republic of Bashkortostan, Russia; The Bonch-Bruevich Saint Petersburg State University of Telecommunications, St. Petersburg, Russia; St. Petersburg State University, Biological Faculty, St. Petersburg, Russia; All-Russian Research Institute for Agricultural Microbiology, Pushkin, St. Petersburg, Russia; All-Russian Research Institute for Agricultural Microbiology, Pushkin, St. Petersburg, Russia

**Keywords:** arbuscular mycorrhiza, SWEET, sugar transport, sucrose, glucose, sugar transporter genes, арбускулярная микориза, SWEET, транспорт сахара, сахароза, глюкоза, гены транспортеров сахаров

## Abstract

Plant sugar transporters play an essential role in the organism’s productivity by carrying out carbohydrate transportation from source cells in the leaves to sink cells in the cortex. In addition, they aid in the regulation of a substantial part of the exchange of nutrients with microorganisms in the rhizosphere (bacteria and fungi), an ty essential to the formation of symbiotic relationships. This review pays special attention to carbohydrate nutrition during the development of arbuscular mycorrhiza (AM), a symbiosis of plants with fungi from the Glomeromycotina subdivision. This relationship results in the host plant receiving micronutrients from the mycosymbiont, mainly phosphorus, and the fungus receiving carbon assimilation products in return. While the eff icient nutrient transport pathways in AM symbiosis are yet to be discovered, SWEET sugar transporters are one of the three key families of plant carbohydrate transporters. Specif ic AM symbiosis transporters can be identif ied among the SWEET proteins. The survey provides data on the study history, structure and localization, phylogeny and functions of the SWEET proteins. A high variability of both the SWEET proteins themselves and their functions is noted along with the fact that the same proteins may perform different functions in different plants. A special role is given to the SWEET transporters in AM development. SWEET transporters can also play a key role in abiotic stress tolerance, thus allowing plants to adapt to adverse environmental conditions. The development of knowledge about symbiotic systems will contribute to the creation of microbial preparations for use in agriculture in the Russian Federation.

## Abstract (ru)

В продуктивности растений существенную роль играют транспортеры сахаров, поскольку с их помощью координируются и осуществляются потоки углеводов от листьев к органам потребления. Кроме того, с участием транспортеров сахаров регулируется значительная часть обмена питательными веществами с микроорганизмами ризосферы (бактериями и грибами), что является необходимым условием для формирования симбиотических отношений. В связи с этим в обзоре уделено особое внимание углеводному питанию при развитии арбускулярной микоризы (АМ) – симбиоза растений с грибами подотдела Glomeromycotina, в результате которого растение-хозяин получает от микосимбионта микроэлементы, главным образом фосфор, а гриб взамен получает продукты ассимиляции углерода. Пути эффективного транспорта питательных веществ в АМ-симбиозе до сих пор не раскрыты. Одно из трех ключевых семейств углеводных транспортеров растений – SWEET, переносчики сахаров. Именно среди белков SWEET могут быть выявлены специфические для симбиоза с АМ-грибами транспортеры. В обзоре представлены данные по истории изучения, структуре, локализации, филогении и функциям белков SWEET. Отмечена высокая вариабельность как самих белков SWEET, так и их функций. При этом одни и те же белки у разных растений могут выполнять различные функции. Особая роль уделена участию транспортеров семейства SWEET в развитии АМ-симбиоза растений и грибов. Транспортеры SWEET могут также играть ключевую роль в устойчивости к абиотическим стрессам, позволяя растениям адаптироваться к неблагоприятным условиям окружающей среды. Развитие знаний о симбиотических системах будет способствовать созданию микробных препаратов для использования в сельском хозяйстве Российской Федерации.

## Introduction

Sugar transporters in plants are customarily divided into three major classes: SUT (SUC), MST (including STP, TMT, PMT, VGT, pGlct/SGB1, ESL, and INT subclasses), and SWEET (Sugars Will Eventually be Exported Transporters). The most well studied transporters are SUT and MST. SUT carry out long-distance transportation of sucrose from plant leaves to the targeted plant organs and tissues. They then disintegrate into monosaccharides and are subsequently transported by MST proteins. The major part of the transporters from SUT and MST classes are known to be non-specific to symbiotic plant-microbial systems such as arbuscular mycorrhiza (AM).

However, in 2010, Li-Qing Chen described a new transporter class SWEET. SWEET transporters carry out non-volatile bidirectional transportation of sugars in all plant organs and tissues. At present, the SWEET protein family is the least studied group of transporters. According to current knowledge, proteins specific to AM symbiosis may be detected inside the SWEET transporter group (Chen et al., 2010). Various sources in the literature provide conflicting information concerning the SWEET protein class. This survey is an attempt to tackle the issue and combine knowledge about the proteins of the group. Thus, the aim of the current research is to provide an overview of the data on gene phylogenesis inside the SWEET class and functions of the proteins encoded with the aforementioned genes as well as to assess their role in the sugar transportation process during the formation of AM symbiosis

## General information on the SWEET transporters

SWEET proteins, identified in the late 1990s, were first called MtN3 (involved in the development of Medicago truncatula Gaertn. nodules) and Saliva (first discovered in the salivary glands of Drosophila during embryonic development). That is why the transmembrane domains made of those proteins were named “MtN3/Saliva”, or “MtN3_slv domain” and are also known as “PQ loop” (Chen et al., 2010). In 2010, Li- Qing Chen was the first to isolate SWEET transporters into a separate family of proteins by providing a detailed description of those pertaining to Arabidopsis thaliana (L.) Heynh. Seventeen different transporters were discovered, described, and named according to the species belonging to a particular plant and the protein number (e. g. AtSWEET17). The same paper provides a detailed description of the SWEET proteins in Oryza sativa L. (Chen et al., 2010). Afterwards SWEET proteins were found in a number of other plant species and also in animals and prokaryotes (the latter were named SemiSWEET) (Chen et al., 2012; Feng et al., 2015; Patil et al., 2015; Manck-Götzenberger, Requena, 2016; Hu L.P. et al., 2017). Now all living organisms are generally considered to possess SWEET or SemiSWEET proteins (Feng et al., 2015). Newly found SWEETs are numbered according to orthology with Arabidopsis (A. thaliana) proteins. However, some discrepancies and variations in A. thaliana numbering have been noted (Supplementary Material 1)1 (Doidy et al., 2019).

## Structure and localization of the SWEET proteins on membranes

SWEET proteins are uniporters, transporting carbohydrates across membranes along a concentration gradient, typically localized on the plasma membrane (Chen et al., 2010). SWEET proteins in plants usually contain seven transmembrane (TM) helices (Xuan et al., 2013). Nevertheless, in 2015, G. Patil et al. discovered that Vitis vinifera L. SWEET is made of fourteen TMH (transmembrane helices), which proves that the SWEET protein helix structure may vary (Patil et al., 2015). Bacterial SemiSWEETs are the smallest among the known transporters. Consisting of about one hundred amino acids coiled in three spirals, i. e., three TMHs, they form a triple helix bundle (THB). The duplication of THB in prokaryotes leads to the emergence of eukaryotic SWEET transporters consisting of two THBs and an additional linker helix, numbered TMH4 (Feng et al., 2015). Moreover, it is noteworthy that the three TMHs in THB are not arranged in series on the membrane. The third TMH is squeezed between the first and the second one. There, the N-terminus of the protein is found on the outer side of the membrane, while the C-terminus is located on the inner side. In eukaryotes, the C-terminus is elongated and contains phosphorylation sites that can be used for posttranslational modification (Jeena et al., 2019). The nucleotide sequence, encoding TMH4, is known to be the most variable of TMHs, and its origin is currently being debated (Jeena et al., 2019).

Supplementary Materials are available in the online version of the paper: http://vavilov.elpub.ru/jour/manager/files/Suppl_Kryukov_Engl


Interestingly, some eukaryotes exhibit SWEET protein structures similar to the prokaryotic ones. For instance, wheat Triticum aestivum L. may also have a SemiSWEET consisting of three and four TMHs (Gautam et al., 2019). According to researchers, the presence of TaSWEET with 3, 4, 6, and 7 TMHs in wheat implies that both duplication and fusion of SWEET protein structures can occur in the genome (Gautam et al., 2019). SuperSWEET sugar transporters found in Phytophthora contain from 18 to 25 TMHs and are composed of 5–8 semiSWEET loops (Jia et al., 2017). Thus, the structures of SWEET proteins should be assumed to be highly variable.

## Phylogenetics of the SWEETs, isoforms

In the construction of a phylogenetic tree, it was revealed that the SWEET genes of plants are still grouped into four clades, despite their low homology (Chen et al., 2015). This evolutionary division occurred long ago, and representatives of each of the clades are observed in almost all (possibly all) terrestrial plants. With all this, the second clade is the most ancient, and its representatives share certain homology with the SWEET proteins in algae (Li X. et al., 2018). In mammals and some microorganisms (e. g. Chlamydomonas), proteins have been found to fall into clade V, separate from the other SWEETs (Chen et al., 2012).

The increasing number of SWEET isoforms is a consequence of duplication or fusion of the THB genes. This contributes to the expansion of transporter functions and plant adaptation under new conditions (Li X. et al., 2018). The number of SWEET isoforms varies significantly among plant species. For example, unicellular and green algae have 1 to 3 SWEET isoforms, while monocots are observed to possess from 18 to 23, and dicots from 15 up to 68 (Li X. et al., 2018). According to other data, T. aestivum wheat (monocotyledonous) is known to have 108 isoforms of SWEET genes localized on 21 chromosomes, while some of them are orthologs of the SWEETs of Arabidopsis (of 14 genes of Arabidopsis), and some belong to three new types that do not have significant homology with Arabidopsis genes (Gautam et al., 2019). M. truncatula has up to 26 isoforms (Doidy et al., 2019). At the same time, it is likely that these are not all of the identified transporters, since, by 2015, only 24 of them were isolated (Chandran, 2015).

The representatives of the four clades are considered to be divided not only phylogenetically, but also functionally. Thus, most researchers argue that (1) the protein representatives of clades I and II transport hexoses, (2) proteins of clade III are mainly involved in the transport of sucrose, and (3) those of clade IV are principally involved in the transport of fructose (see Suppl. Material 1) (Chen et al., 2012; Feng et al., 2015). But this is not necessarily so. In 2019, B. Hu et al. showed that MtSWEET5b and MtSWEET7 (M. truncatula) are able to transport not only hexoses, but also sucrose. Other plants may also be exceptions, for instance LjSWEET3 (Lotus japonicus L.) also transports sucrose instead of hexoses. MtSWEET16 may be involved in the transportation process of sucrose and mannose. It is therefore impossible to speak strictly about the clade division of the SWEET genes for the types of the transferred substrate (see Suppl. Material 1) (Hu B. et al., 2019).

The nucleotide sequence analysis of the SWEET genes shows their significant variability. Between the four clades, it can reach up to 80 % (which is to say that, in some cases, there is homology of only 20 %) (unpublished data, Kryukov et al., 2021). With such variability, it is typically impossible to align sequences and then build phylogeny. In this regard, the existing phylogenetic trees of the SWEET genes should be treated with extreme caution. The intron-exon structure of the SWEET genes may also vary notably (Cao et al., 2019). Most of the MtSWEET genes (in M. truncatula) contain 5 introns, excluding the genes MtSWEET4, MtSWEET6, MtSWEET7 and MtSWEET13 which include 4 introns, and MtSWEET2b which contains 16 introns (Hu B. et al., 2019). The structure of the M. truncatula SWEET proteins is also heterogeneous: most contain 7 TMHs, but MtSWEET4 and MtSWEET11 have 6 TMHs, and MtSWEET2b contains 15 TMHs instead of 7 (Hu B. et al., 2019).

## SWEET protein functions

As has already been mentioned, the representatives of the four clades can be divided in accordance with their functions. However, it should be noted that different authors provide varied data on functions of the certain SWEET proteins (see Suppl. Materials 1 and 2). This may be due to several possible reasons: (1) orthologs of the SWEETs can perform different functions in different species; (2) orthologs can perform different functions under different conditions and their genes are expressed in different ways; (3) possible paralogs within each clade may be similar and hence may be misidentified.

In all cases, SWEET proteins are non-volatile bidirectional uniporters. However, according to some researchers, the fact that all SWEET transporters are uniporters has not been completely proven (Chen et al., 2015). SWEET proteins are involved in a variety of processes, whether in plants (see Suppl. Material 1) or mammals. In addition to the transportation of carbohydrates, they are most likely to participate in the transport of other agents such as gibberellins, which is the case of Arabidopsis (Kanno et al., 2016). In peas (Pisum sativum L.), it was also discovered that the interaction between the SWEET transporters and CWINV (cell wall invertase) in the presence of cytokinins leads to the formation of multiple shoots and the loss of apical dominance during infection with the pathogen Rhodococcus fastian (Doidy et al., 2019).

SWEET transporters can also play a role in abiotic stress tolerance, allowing plants to adapt to adverse environmental conditions (see Suppl. Materials 1 and 2) (Chandran, 2015). Various authors have associated the accumulation of sugars in plants with abiotic stresses (Hu B. et al., 2019). Low temperatures, water, and other stressful environmental factors are able to induce the expression of the SWEET genes in plants, which leads to the assumption that these genes are associated with plant responses to these stresses (Kafle et al., 2019; Wei et al., 2020).

There is a great deal of literary data on the functions of the SWEET proteins in plants of various species. For example, LjSWEET3 mediates the transportation of sucrose (Sugiyama et al., 2017) to nodules. The AtSWEET1 and AtSWEET5 genes are significantly expressed at different stages of pollen maturation. Almost all representatives of clade II are involved in the transportation of sugars to the reproductive organs, i. e., pollen, seeds, and some to fungal pathogens (Chen et al., 2010). Genes AtSWEET11 and AtSWEET12 have been established as important transporters of sucrose from parenchyma cells to phloem (Chen et al., 2012). At the same time, SWEET proteins of the clade III are associated with susceptibility and resistance to pathogens (Gautam et al., 2019). According to W.J. Guo et al., proteins of the clade IV – AtSWEET17, AtSWEET16 – are active in root cortical cells and are localized on the tonoplast (Guo et al., 2014).

Rhizosphere pathogens can cause an increased expression of clade III proteins, which leads to additional transport of sucrose to the roots and contributes to the nutrition of rhizosphere microorganisms (Doidy et al., 2019). In 2010, it was shown by L.-Q. Chen et al. that pathogenic bacteria, for example Xanthomonas, are able to enter tissues of the host plant and induce the expression of SWEET genes (primarily SWEET11 and SWEET14, from clade III) to obtain sugars. Like symbiotic AM fungi, pathogenic fungi also have the ability to induce the expression of genes in order to get sugar for themselves (Chen et al., 2010).

The expression of a significant number of alterations in SWEET under the influence of stress factors such as water deficiency leads to a notably increased expression of the MtSWEET3a, MtSWEET3b, MtSWEET9b and MtSWEET13 genes, while the expression of MtSWEET1a, MtSWEET3c, MtSWEET15c drops significantly (see Suppl. Material 2) (Hu B. et al., 2019). According to J. Doidy et al., MtSWEET16 is unique in that its expression is mainly enhanced in leaves, whereas the pea ortholog PsSWEET16 is expressed primarily in the roots and stem (Doidy et al., 2019). SWEET3 orthologs PsSWEET3.1, MtSWEET3.3 and LjSWEET3 (Sugiyama et al., 2017), SWEET11 orthologs MtSWEET11 and PsSWEET11 (Kryvoruchko et al., 2016) and SWEET15 orthologs MtSWEET15.3 and PsSWEET15.3 (Gamas et al., 1996) are specifically expressed in root nodules in leguminous plants. J. Manck-Götzenberger and N. Requena note that numerous transporters show significant expression in AM symbiosis while being non-specific to it (Manck-Götzenberger, Requena, 2016). In turn, A. Kafle pointed out that SWEET1 orthologs (MtSWEET1.2 and PsSWEET1.2) can be expressed in both mycorrhized roots and root nodules (Kafle et al., 2019).

## Localization and functions of the SWEET transporters in root cells of plants with arbuscular mycorrhiza fungus

As is known, according to the data of the transcription profiles, not all transporters of the SWEET family have yet been found, nor have all known transporters of this group been localized in a plant cell and their exact function established (Hennion et al., 2019). Only now is the localization of most SWEET sugar transporters receiving proper attention (see Suppl. Materials 1 and 2) as such study requires a separate examination of each individual transporter for each individual plant species. Their functions and localization also require confirmation. On the other hand, the question of the participation of SWEET proteins in the specific transport of sugars from the host plant to mycosymbiont AM fungi is quite urgent and requires detailed research, since knowledge of the mechanisms of active carbohydrate nutrition of a mycosymbiont will allow us to understand the mechanisms that lead to the formation and development of effective interaction between partners in AM symbiosis.

According to the literary data, it should be assumed that most of the SWEET transporters of the clades I, II, III are localized in the plasma membrane (see the Figure). The figure represents a root cell of a host plant with an arbuscule (arbuscule is the most common type of symbiotic structure formed during the development of AM; this is the invagination of plant plasmalemma into the plant cell at the site of penetration of the AM fungus hypha, followed by multiple branching of the trunk of the arbuscule with the formation of a new interface for the interaction of symbiotic partners – the periarbuscular space (PAS) – between the periarbuscular membrane (PAM) and the arbuscular membrane (ArM) with the arbuscular cell wall (ACW), formed in place of the cell wall of the host plant).

The peculiarity of transport processes under AM conditions is analyzed by comparing cells with and without arbuscules (Gaude et al., 2012). Thus, J. Manck-Götzenberger and N. Requena (Manck-Götzenberger, Requena, 2016) were the first to show that the main transport of sugars in Solanum tuberosum from the host plant to the AM fungus Rhizophagus irregularis can occur due to the facilitators of sucrose and glucose – StSWEET12 and StSWEET7a, respectively ( 1 , see the Figure; Manck-Götzenberger, Requena, 2016; Hennion et al., 2019). StSWEET12 and StSWEET7a operate on PAM and transport sugars from the cytoplasm to the PAS and vice versa. From here, glucose is transported through the ArM from PAS in the arbuscule using the fungal monosugar transporter RiMST2 (R. irregularis (Błaszk., Wubet, Renker & Buscot)) (Hennion et al., 2019), or as a result of GpMST1 functioning (Geosiphonomyces pyriformis Cif. & Tomas) (Schüßler et al., 2006). The sucrose transportation through the ArM may occur via the fungal sucrose transporter RiSUC1 (Helber et al., 2011). Subsequently, sugar is transported along the intraradical mycelium as glycogen into the extraradical mycelium of the AM fungus (Hennion et al., 2019). On the other hand, the cytoplasm sugar in the cells of the root cortex can be regulated by their transfer from the vacuole by tonoplastic transporters, which include the glucose facilitator StSWEET2c ( 2 ; Hennion et al., 2019).

The sugar transportation apoplastic pathway is carried out to cells both with and without AM fungus via SWEET hexose transporters ( 3 ; Chardon et al., 2013; Ludewig, Flügge, 2013). It is assumed that there may be specific SWEET facilitators for AM symbiosis. For example, StSWEET12 and StSWEET7a proteins may carry out specific transportation of sucrose and glucose in S. tuberosum through the plasmalemma of root cortex cells containing arbuscules ( 4 , Manck-Götzenberger, Requena, 2016; Hennion et al., 2019). Once there, the effectors secreted by AM fungi either directly activate the expression of the SWEET genes, or indirectly through the activation of transcription factors ( 5 ; Chandran, 2015; Jeena et al., 2018). The LjSWEET3 protein, which is responsible for the transportation of sucrose to the cells with arbuscules in Lotus japonicus, is claimed to be a specific facilitator as well ( 6 , see the Figure; Sugiyama et al., 2017; Hennion et al., 2019). Proteins AtSWEET15 (previously called SAG29; Seo et al., 2011) and MtSWEET11 (Kryvoruchko et al., 2016) are known to be non-AM-specific SWEET transporters, localized on the root cell plasma membrane ( 7 and 8 , respectively).

**Fig.**
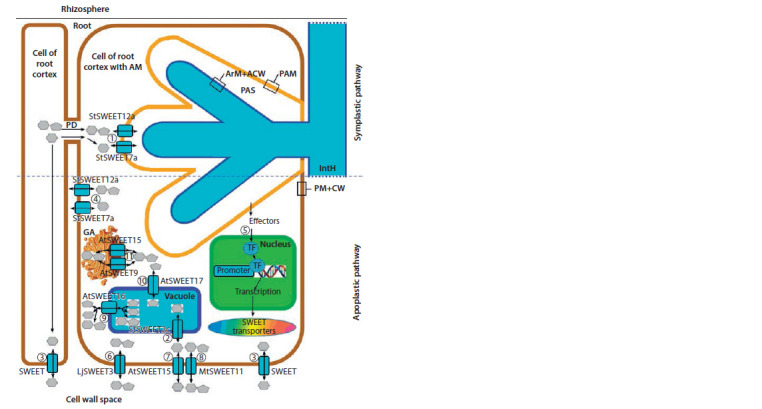
Localization scheme of the SWEET transporters in a cell with an arbuscule (Guo et al., 2014; Lin et al., 2014; Chandran, 2015; Chen et al., 2015; Ait Lahmidi et al., 2016; Kryvoruchko et al., 2016; Manck-Götzenberger, Requena, 2016; Sugiyama et al., 2017; Hennion et al., 2019; Jeena et al., 2019; Yurkov et al., 2019). PM + CW – plasmalemma and cell wall of the root cortex; PAM – periarbuscular membrane; PAS – periarbuscular space; ArM + ACW – arbuscular membrane and arbuscular cell wall; IntH – intercellular intra-root hypha of AM fungus; TF – transcription factor; GA – Golgi apparatus; PD – plasmodesmata. Description of the circuit is provided in the main article.

The discussion of the SWEET protein localization on the organelles of the root cell is controversial. Thus, according to some data, transporters of the clade IV (AtSWEET16 and AtSWEET17) can be localized in the tonoplast of the plant vacuole ( 9 and 10, respectively; Chardon et al., 2013; Guo et al., 2014; Jeena et al., 2019). On the other hand, clade III sucrose transporters AtSWEET9 and AtSWEET15 may be localized on the membrane of the trans- Golgi network (11 , see the Figure).

Thus, summarizing the information on the localization of the SWEET transporters in AM, it can be concluded that none of the transporters has shown specific localization simultaneously in two or more plant species. Nor is there attested specific gene expression under the same conditions, as, for example, in the phosphate transporter (PT4) of M. truncatula and in a number of other plant species. The first to be verified are StSWEET12 and StSWEET7a.

The transporter functions in AM may be assumed on the basis of general information about the clades of proteins of the SWEET family, but it should be noted that there have been no detailed studies of both the localization and functions of these proteins in AM symbiosis yet. There are only assumptions about their role in AM. For instance, in a recent work by J. An et al. (2019), it has been noted that MtSWEET1b may supply AM glucose to fungi. According to the M. truncatula gene expression atlas (MtGEA; http://mtgea.noble.org/v3/), MtSWEET1b and MtSWEET6 are highly expressed in arbuscular cells, and their putative orthologs StSWEET1a, StSWEET1b, and StSWEET7a (S. tuberosum L.) also demonstrate high transcription levels in mycorrhizal roots (Manck-Götzenberger, Requena, 2016). SWEET transporters of clade I are those most likely to participate in the supply of sugars to symbiotic systems, including AM (Doidy et al., 2019). Based on this information, it should be assumed that studies of the function of the SWEET proteins are still very fragmentary (see Suppl. Material 1). The confirmation in several plant species remains an urgent task.

## Conclusion

SWEET proteins are essential for the transportation of carbohydrates in plants. Proteins specific to the various forms of symbiosis can be found amongst the SWEET class. Primarily, they can be located in clades I and III. SWEET transporters are quite variable, a change in external conditions may lead to the emergence of numerous isoforms with varying functions. Hence, SWEET protein identification and selection of primers for the gene amplification requires prudence. Close paralogs may be very similar; however, high variability between clades does not allow for the construction of a reliable phylogenetic tree with all the ensuing consequences. This high variability may account for the scatter of the data related to SWEET protein functions (see Suppl. Material 1). Still, a hypothesis about the universality of the range of SWEET genes may be put forward, mainly in case of similar gene structure. Furthermore, there are reasons to believe that not all of the genes from the SWEET class have yet been identified for M. truncatula. All this testifies in favor of the fact that the understanding of the functions of these transporters will be expanded in the coming years.

## Conflict of interest

The authors declare no conflict of interest.
